# Outcomes of High-Dose Inhaled Nitric Oxide and Oxygen Administration for Severe Pulmonary Hypertension With Bronchopulmonary Dysplasia

**DOI:** 10.7759/cureus.58855

**Published:** 2024-04-23

**Authors:** Midori Takahashi, Masahiko Murase

**Affiliations:** 1 Children Medical Center, Showa University Northern Yokohama Hospital, Kanagawa, JPN

**Keywords:** bronchopulmonary dysplasia, extremely low birth weight infant, pulmonary hypertension, sildenafil, nitric oxide

## Abstract

Pulmonary hypertension (PH) with bronchopulmonary dysplasia (BPD) is fraught with high infant mortality rates. However, the intervention strategy for severe PH is unclear. This case report discusses the utility of long-term high-dose inhaled nitric oxide (iNO) administration and that of oxygen therapy for the prevention of PH deterioration.

A male infant weighing 864 g was delivered at a gestational age of 24 weeks and three days. The patient who had severe BPD was diagnosed with PH at a corrected gestational age (CGA) of 43 weeks. Although oxygen was administered to prevent PH, the patient still developed severe PH. Despite long-term high-dose (iNO) administration, the patient could not survive. The abovementioned treatment may exacerbate PH, and oxygen administration is less effective for the prevention of PH deterioration with BPD.

## Introduction

Pulmonary hypertension (PH) with bronchopulmonary dysplasia (BPD) is fraught with high infant mortality rates. Many intervention strategies for PH with BPD have attempted to reduce the mortality rate; however, evidence of mortality rate reduction is still scarce [1-3]. Although the approved maximum dose of inhaled nitric oxide (iNO) is 20 PPM in Japan, we experienced a case that used the maximum dose of 70 PPM in the long term. No case report reported the use of long-term high-dose iNO. This case report illustrates one attempt at an intervention strategy for PH with BPD and histopathological findings. Herein, we discuss the utility of long-term high-dose iNO administration for severe PH with BPD and that of oxygen therapy for the prevention of PH deterioration.

## Case presentation

**Figure 1:**
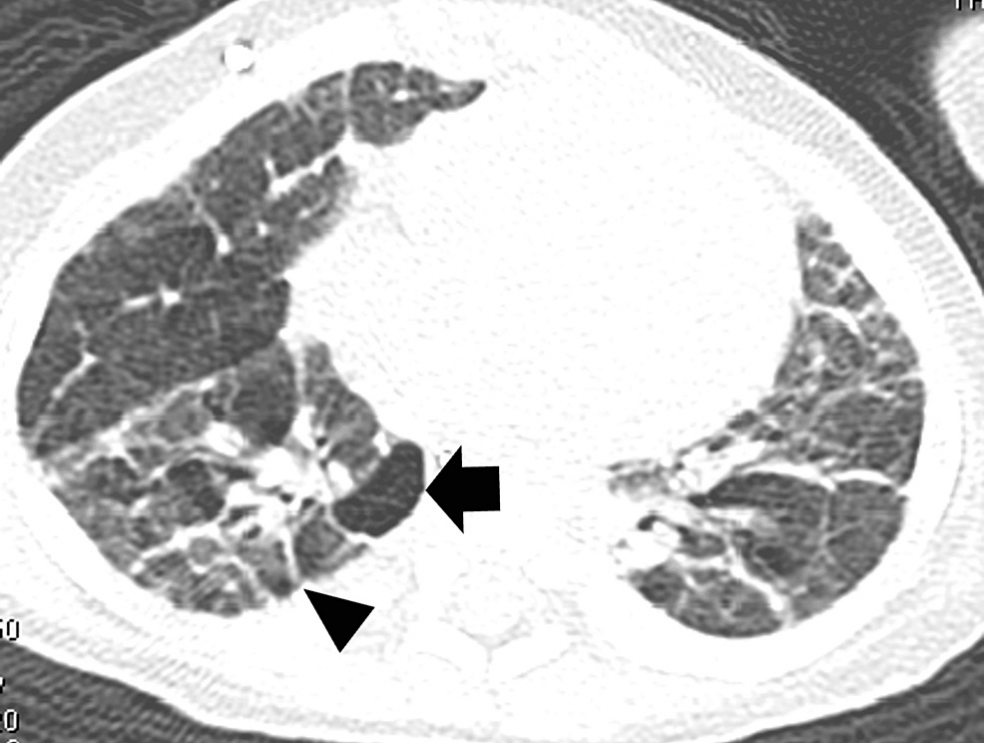
CT finding before PH crisis Cystic change is shown in the black arrow. Fibrotic change is shown in the black head. CT, computed tomography; PH, pulmonary hypertension

A male infant weighing 864 g was delivered vaginally at a postmenstrual age of 24 weeks and three days due to preterm labor and subsequent tocolytic failure. He received mechanical ventilation until a corrected gestational age (CGA) of 40 weeks one day, followed by nasal continuous positive airway pressure (CPAP) until 43 weeks and six days of CGA. Then, he was diagnosed with severe BPD. A computed tomography (CT) scan performed at 56 weeks and two days of CGA revealed findings consistent with BPD (Figure 1). His condition was complicated by intraventricular hemorrhage, patent ductus arteriosus, sepsis, retinopathy of prematurity, bronchomalacia, and gastroesophageal reflux.

On 43 weeks of CGA, an echocardiogram showed ventricular septal flattering, right ventricular hypertrophy, and pulmonary artery (PA) dilation. The peak tricuspid regurgitant velocity, as revealed by echocardiography, and estimated PA pressure were 3.3 m/s and 42 mmHg, respectively, obtained by the simplified Bernoulli equation. He was diagnosed with PH. Thirty percent oxygen administration and 3L/min of flow rate by nasal cannula continued after nasal CPAP were discontinued. At 57 weeks of CGA, the echocardiogram revealed worsening PH and increased pulmonary arterial pressure. Despite the increased oxygen concentration, oxygenation deteriorated. At 59 weeks and two days of CGA, sildenafil therapy was initiated at a dose of 0.1 mg/kg/day and increased to 0.2 mg/kg/day.

**Figure 2:**
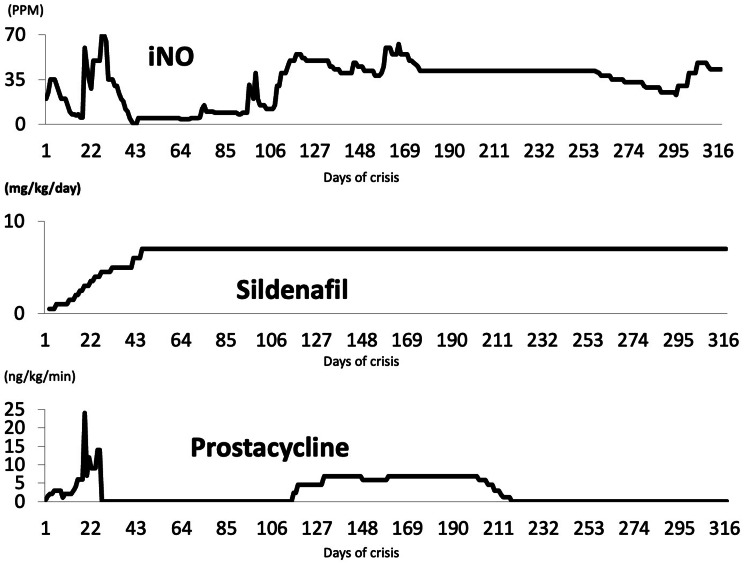
Course of drug administration The course of drug administration is shown. Since the milrinone dose of 0.5 ug/kg/minute was not changed and it was administered for 15 hours, we did not show the milrinone trend graphically.

At 60 weeks of CGA, following a bout of emesis, he acutely developed tachycardia and hypoxemia. Blood gas showed a pH of 7.08 with a pCO_2_ of 69.5 mmHg. The echocardiogram did not show the tricuspid regurgitant jet velocity but revealed that the intraventricular septum bowed into the left ventricle at end-systole. Since the abovementioned finding indicated that pulmonary pressure exceeded systemic pressure, severe PH was diagnosed on the first day of crisis (DOC) [4]. On the second DOC, the treatment plan to stabilize PH was adjusted to include sedation, mechanical ventilation, and the administration of epoprostenol, iNO, and sildenafil (drug administration courses are shown in Figure 2). The high dose of iNO was gradually reduced. However, on the 19th DOC, due to severe PH, the iNO doses were increased. In addition, epoprostenol doses were also increased, leading to transient stabilization. The infant’s condition allowed a reduction in the dose of epoprostenol. However, attempts at weaning iNO were not tolerated. On the 25th DOC, epoprostenol was increased to reduce the iNO dose. On the 25th DOC, epoprostenol was increased and milirinoe therapy was initiated to reduce the iNO dose. On the 26th DOC, the sildenafil dose was increased. On the same DOC, his blood pressure decreased from 113/68 mmHg to 59/44 mmHg and his peripheral pulse was not palpated. Echocardiography revealed a low left ventricular volume. Because we diagnosed the patient with hypovolemic shock, fluid resuscitation was initiated, leading to blood pressure recovery. He possibly developed hypovolemic shock due to peripheral vasodilation by vasodilator. Thus, the iNO dose was increased to 70 PPM with the approval of the patient’s parents, and milrinone and epoprostenol were discontinued.

On the 32nd DOC, the sildenafil dose was increased, and iNO was tapered off as the PH stabilized. On the 109th DOC, severe PH necessitated iNO dose increments. Although prostacyclin intravenous administration was initiated, the infant did not respond to this medication. The infant developed multifocal pneumothorax on the 284th DOC, which did not improve with chest tube placement. On the 317th DOC, he expired secondary to tension pneumothorax.

Methemoglobin forms as the result of hemoglobin reacting with the iNO that diffuses into the bloodstream at the alveolar-capillary interface, and its concentration is elevated in proportion to the iNO administration dose [5]. However, in this case, The methemoglobin concentration ranged from 0.5 to 2.8 during the PH crisis and from 0.5 to 2.2 after prostacyclin intravenous administration and the concentration of methemoglobin did not increase. This clinical course suggests that iNO diffusion was prevented due to the patient’s ventilation-perfusion mismatch.

**Figure 3:**
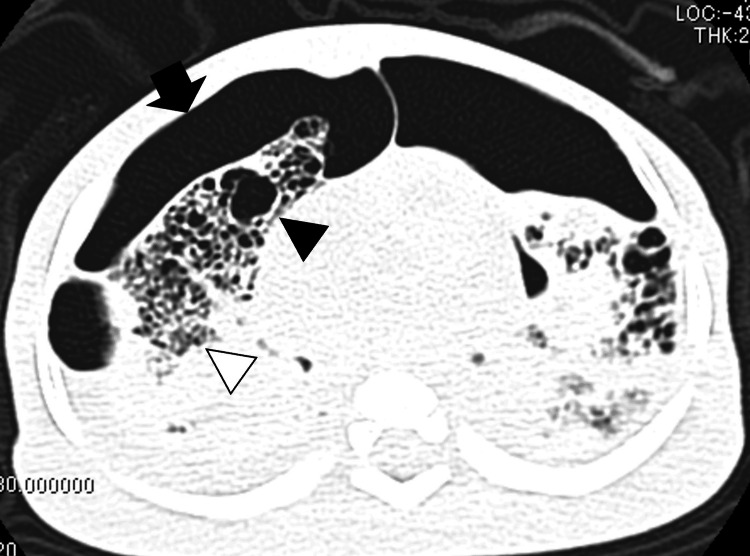
Postmortem CT findings The air leak is shown in the black arrow. Cystic change is shown in the black head. Fibrotic change is shown in the white head. CT, computed tomography

**Figure 4:**
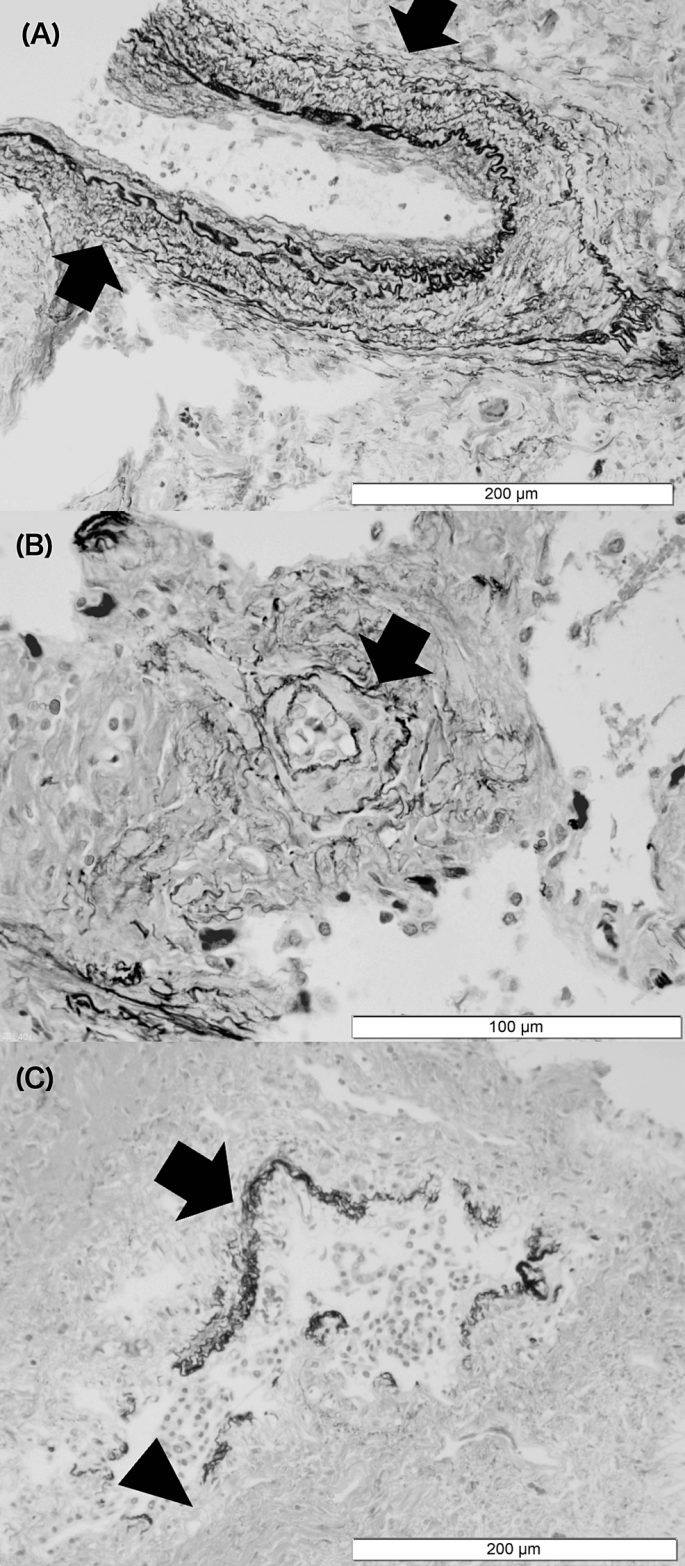
Lung histopathology (A, B) Medial hypertrophy in the small pulmonary artery is shown in the black arrow. (C) The respiratory bronchiole wall is thick, as shown with the black arrow. This figure does not show alveoli, and fibrotic changes are only shown around respiratory bronchioles (black head). Since all figures show scales, magnification is not shown.

The parents consented to lung tissue needle biopsies and CT performed after he expired (CT and pathological findings are shown in Figures 3, 4). The biopsies showed that small PAs developed medial hypertrophy due to hypoxia (Figures 4A, 4B), a common pathological change with PH that contributes to increased pulmonary vascular resistance. The respiratory bronchiole walls are dilated and thick (Figure 4C). Fibrotic changes were present around the alveoli and respiratory bronchioles, and the alveoli malfunctioned because of these changes.

## Discussion

Herein, we present the case of a patient who developed severe PH with BPD and could not be rescued even using high-dose iNO and oxygen therapy. To resolve PH with BPD, it is necessary to 1) induce vasorelaxation of constricted PAs and improve ventilation-perfusion matching, 2) prevent or attenuate the abnormal remodeling, and 3) promote normal lung development [6]. In this case, PH was stabilized by iNO. The proposed mechanism of action of iNO is not only pulmonary vasodilation and increased compliance but also reduced inflammation and fibrosis [3]. However, the histopathological findings show that abnormal remodeling and disrupted alveolarization could not be promoted in our case. Disrupted alveolarization causes PA hypoxia, inducing vasoconstriction and remodeling vascular structure [1]. As a result, long-term iNO administration exacerbates PH due to mechanical ventilation, even though high-dose iNO administration is effective for the stabilization of severe PH. To the best of our knowledge, there is no known effective treatment for severe PH with BPD. Less use of mechanical ventilation is to prevent PH deterioration [3]. Therefore, long-term and high-dose iNO administration for severe BPD PH may not be recommended if mechanical ventilation settings cannot be changed to less invasive.

Hypoxia is a risk for PH deterioration (3). During the PH with BPD diagnosis, oxygen therapy is typically administered to prevent PH deterioration due to hypoxia. The patient needed high-concentration oxygen to prevent hypoxia, and the patient potentially developed a ventilation-perfusion mismatch. Although the patient was on high-concentration oxygen, severe BPD developed, causing a ventilation-perfusion mismatch, and small pulmonary vessels developed with hypoxia as a result, regardless of oxygen administration due to ventilation-perfusion mismatch. However, high-dose oxygen administration is a risk factor for BPD. Oxygen therapy is limited, and it would not have prevented PH deterioration when there is a ventilation-perfusion mismatch. Another approach for the prevention of PH deterioration is, therefore, needed.

The patient was administered sildenafil to prevent PH deterioration initially only two days before developing severe PH. Sildenafil reduces the PA pressure [3]. A previous meta-analysis shows that sildenafil improves pulmonary arterial pressure within one to six months [7]. In addition, prophylactic sildenafil administration for preterm infants does not induce BPD or death [3]. Sildenafil may require time for PA pressure reduction. There is low-quality evidence to support sildenafil use in preterm infants, especially in the case of severe BPD PH [3]. The ongoing randomized study conducted to clarify the effect of sildenafil effect on BPD PH is expected to provide valuable information on the effect of the drug on BPD PH [8].

Pathological autopsy is the best method to confirm histopathological findings. However, a needle biopsy was performed in this case and provided limited findings. It is the limitation of our case report. However, the CT findings show that BPD developed throughout the lung. Therefore, we think the histopathological findings apply to the entire lung of this patient.

## Conclusions

High-dose iNO and oxygen administration could not rescue patients with severe BPD PH due to mechanical ventilation injury. Therefore, long-term high-dose iNO administration for severe BPD PH may not be recommended. 
